# Hierarchical Structure Kaolinite Nanospheres with Remarkably Enhanced Adsorption Properties for Methylene Blue

**DOI:** 10.1186/s11671-019-2934-x

**Published:** 2019-03-19

**Authors:** Qian Zhang, Yude Zhang, Juntao Chen, Qinfu Liu

**Affiliations:** 1 College of Chemistry and Chemical Engineering, Henan Key Laboratory of Coal Green Conversion, Henan Polytechnic University, Jiaozuo, 454000 China; 2 School of Geoscience and Surveying Engineering, China University of Mining and Technology, Beijing, 100083 China

**Keywords:** Hydrothermal, Kaolinite, Nanospheres, Microstructure, Adsorbent, Methylene blue

## Abstract

Kaolinite nanospheres with hierarchical structures were synthesized via dehydration—rehydration technique through calcined—hydrothermal route. The microstructure of samples were characterized and analyzed by diverse techniques. The results show that after hydrothermal treatment, the layered pseudo-hexagonal kaolinite particles transformed to hierarchical structure nanospheres. The hierarchical structures exhibit large specific surface area of 157.1 m^2^ g^−1^ and narrow mesoporous size distribution. The adsorption properties of kaolinite nanospheres were systematically investigated by the removal of methylene blue (MB) from water. It was found that the nanospheres can rapidly adsorb MB with a higher adsorption capacity (184.9 mg/g), and adsorption data followed Langmuir isotherm model and pseudo-second-order kinetic model. Furthermore, the adsorbent can be regenerated by washing with methanol-HCl solution and shown removal efficiency of more than 95% up to 4 cycles.

## Introduction

Dyes are synthetic aromatic compounds which are widely used in textile, leather, paper, plastic, and other industries [1]. With the development of industry, the water pollution has been realized and gradually become one of the most serious concerns of current age [2]. Lots of remediation methods of polluted wasters include flocculation, precipitation, ion exchange, membrane filtration, electrochemical destruction, irradiation, and ozonation. Adsorption has long been considered as a highly efficient approach for pollution control, and various adsorbents such as activated carbons, fly ash, clay minerals, and metal oxides have been developed for the removal of contaminants from wastewater [3–7].

Kaolinite (Kaol) of chemical formula Al_2_Si_2_O_5_(OH)_4_ is a dioctahedral 1:1 phyllosilicate formed by superposition of silicon tetrahedral sheets and aluminum octahedral sheets [8]. Based on its abundant availability, low cost, and special structure, Kaol has attracted much attention from an environmental perspective as a promising low-cost adsorbent [9, 10]. However, raw Kaol exhibit relatively low sorption capacity due to the low reactivity and specific surface area. Researchers have approved that nanomaterials and nanotechnologies have been shaping the wastewater treatment process unprecedentedly [11–14]. In order to increase the reactivity and specific surface area of Kaol, various methods such as organic modification, acid or alkaline activation, delamination, and exfoliation were developed [15–18]. However, due to the inaccessible interlayer space of kaolinite, these methods need lots of chemical agents and the repeated intercalation-deintercalation or steps displacement intercalation of Kaol for days or weeks to get Kaol nanoparticles [19, 20]. In nature, kaolin group clay minerals are formed through hydrothermal alteration or weathering process. Lots of interest have been paid to the formation of Kaol mineral using aluminosilicate gels as starting material in lab [21–24]. An interesting finding is that the hydrothermal formed Kaol exhibit various morphology nanostructure [25]. In addition, some nanostructure clay minerals such as hydrosodalite [26], nepheline [27], illite [28], metal doped clay minerals [23, 29–31], and tobelite [32] have been manufactured through hydrothermal technology employing kaolin combined with silicic acid, aluminum nitrate, NaOH, kOH, or NH_3_ solution.

Inspired by the above researches, we propose a calcined-hydrothermal combined technique to prepare hierarchical structured nanospheres using Kaol as the starting materials without using any chemical agent. The obtained materials presented a unique hierarchical pomegranate-like kaolinite superstructure (noted as PS-Kaol) composed of numerous kaolinite nanospheres with large specific surface area and abundant mesoporous. Furthermore, the adsorption performance of PS-Kaol was measured by the remove of methylene blue (MB) from water.

## Materials and Methods

### The Aims of the Study

To significantly increase the specific surface area of kaolinite and improve its sorption capacity of dyes from water, the hierarchical structured kaolinite nanospheres were prepared through an environment friendly calcined-hydrothermal combined technique without any chemical agent. To preliminarily evaluate its absorbability, the adsorption performance of PS-Kaol was measured by the removal of MB from water.

### Materials

The sample used in this study was the natural kaolin from Guangxi province of China. Its chemical composition in wt.% is SiO_2_ 49.52, Al_2_O_3_ 35.62, Fe_2_O_3_ 0.62, MgO 0.23, CaO 0.41, Na_2_O 0.36, K_2_O 0.10, TiO_2_ 0.12, P_2_O_5_ 0.86, SO_3_ 0.07, and loss on ignition 12.09. MB was obtained from Tianjin ShengAo Chemical Reagents Company. It is a cationic dye, with the molecular formula C_16_H_18_ClN_3_S·3H_2_O, a molar mass of 373.90 g mol^−1^, and a maximum absorbance equal to 664 nm. The methanol and HCl were purchased from Beijing Chemical Reagents Company, China. The distilled water was used in all experiments.

### Preparation of Hierarchical Kaolinite Nanospheres

The raw Kaolin samples were purified by sedimentation in water to remove the settled residues and then the suspended slurry was spray dried to form ball-like kaolinite aggregation. The purified Kaol powders were then calcined at 600 °C for 2 h in a muffle furnace under air environment to get calcined Kaol (noted as C-Kaol). During this calcined treatment, the Kaol undergoes an important modification and becomes much reactive [33]. The activated Kaol is an important starting material for the followed hydrothermal treatment. Typically, 5 g C-Kaol and 60 ml distilled water were mixed and stirred vigorously for 30 min. Then this mixture was transferred into a 100 ml Teflon-lined stainless steel autoclave and hydrothermally treated at 200 °C under magnetic stirring for a duration of 48 h and cooled to room temperature. Finally, the final product was collected by centrifugation and dried at 100 °C for 10 h.

### Characterization

The morphologies and structures of the samples were observed by scanning electron microscopy (HSEM Hitachi, SU8020) and transmission electron microscopy (TEM, JEM1200EX), respectively. The XRD patterns were recorded using a Bruker D8 instrument with a copper target. Fourier-transform infrared spectra (FT-IR) was recorded in KBr pellets with 2 cm^−1^ resolution on Bruker Tensor 27 spectrometer. X-ray photoelectron spectroscopy (XPS) was carried out on Thermo escalab 250Xi spectrometer. Nitrogen adsorption-desorption isotherm was taken with an Autosorb-iQ-MP analyzer (Quanta Chrome, USA).

### The Adsorption Experiments

The adsorptive capacity of the samples was evaluated using MB as typical indicator. A series of adsorption experiments with varying contact time, pH, initial concentration of MB, and recycling were conducted to investigate the adsorption capacity of adsorbents. Typically, 100 mg of adsorbent was mixed with 100 ml MB aqueous solution with various concentrations in 250 ml conical beaker by magnetic stirring at 25 °C for a certain time. The influence of contact time was tested from 5 to 120 min (at 25 °C, initial pH value ~  6.5, MB 100 mg/L). To evaluate the pH effect, the range of 2 to 12 was selected (contact time: 12 h at 25 °C, MB 100 mg/L) and solution pH was adjusted by adding HCl and NaOH (0.1 mol L^−1^). The concentration of 50, 80, 100, 150, 200, 300, and 400 mg/L were selected to study the effects of initial MB concentration (at 25 °C, initial pH without adjustment, 12 h). In order to investigate the recyclability of the absorbents, the powders were collected; after that, the adsorption reached the equilibrium in MB solution at 100 mg/L at 25 °C and initial pH value. Then they were washed with the methanol-HCl mixed solution for deabsorption. After separated and dried, the samples were reused to adsorb MB. The regeneration and resorption process was repeated four successive cycles. For each adsorption test, the solution was taken and centrifuged to remove the adsorbent. The supernatant of the centrifuged solution was analyzed by a DR2800 water quality analyzer (HACH, America). The adsorption performance is evaluated using the following expressions:

1 $$ \mathrm{Adsorption}\ \mathrm{percentage}=\frac{C_0-{C}_{\mathrm{e}}}{C_0}\times 100\% $$

2 $$ {q}_{\mathrm{e}}\left(\mathrm{mg}/\mathrm{g}\right)=\frac{\left({C}_0-{C}_{\mathrm{e}}\right)V}{m} $$

3 $$ {q}_{\mathrm{t}}\left(\mathrm{mg}/\mathrm{g}\right)=\frac{\left({C}_0-{C}_{\mathrm{t}}\right)V}{m} $$

where *C*_0_ (mg/L) is the initial MB concentration, *C*_e_ (mg/L) is the equilibrium MB concentration, *C*_t_ (mg/L) is the MB concentration in the aqueous solution at time *t* (min), *q*_e_ (mg/g) is the equilibrium adsorption capacity, *q*_t_ (mg/g) is the adsorption capacity at time *t* (min), *V* (L) is the volume of the solution, and *m* (g) is the mass of the adsorbent.

## Results and Discussion

### Material Characterization

**Fig. 1:**
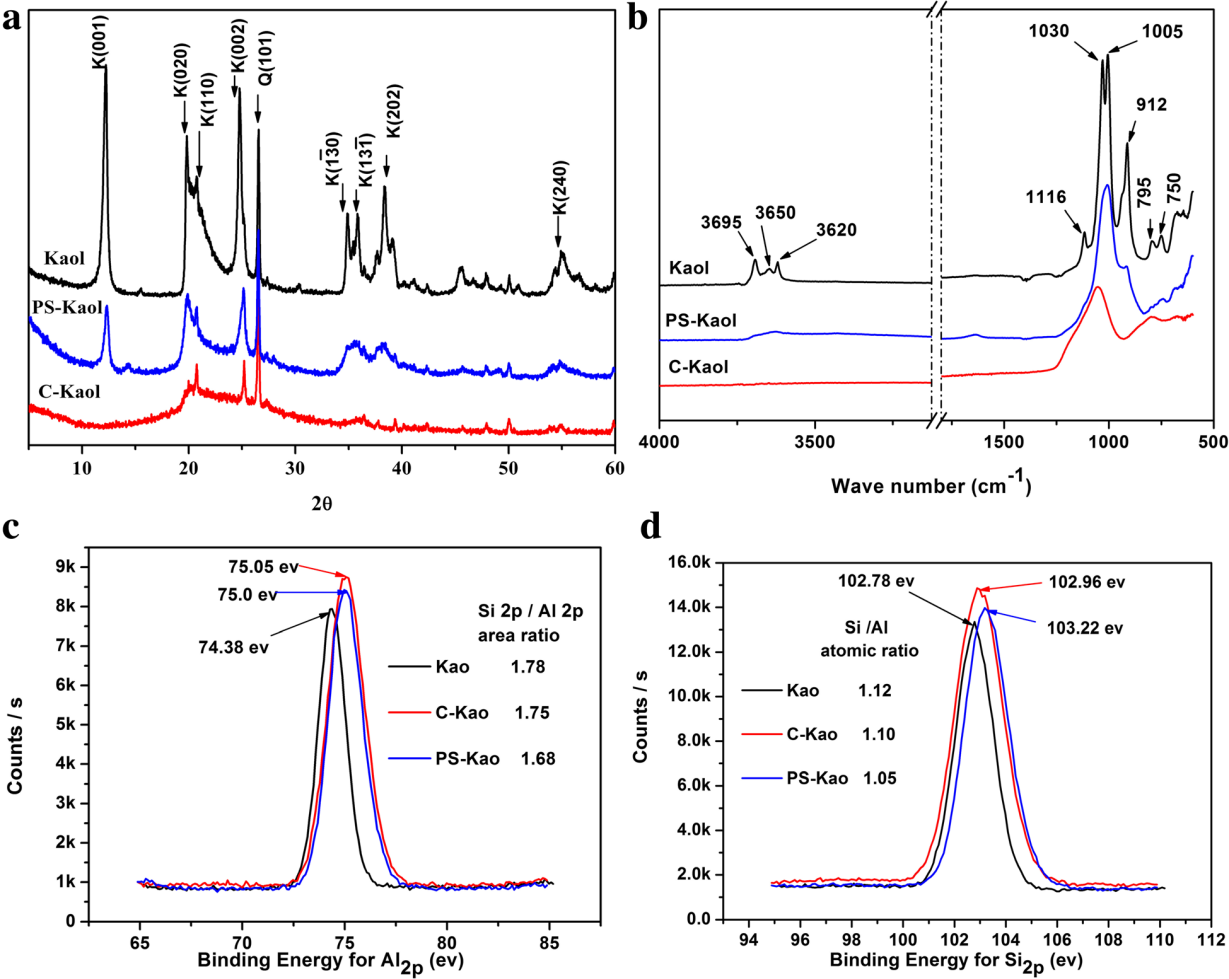
**a** XRD patterns, **b** FTIR spectra, **c** Al_*2p*_ XPS spectra, and **d** Si_*2p*_ XPS spectra of the samples Kaol, calcined Kaolinite (C-Kaol), and the hydrothermal treatment calcined Kaolinite (PS-Kaol)

Figure 1a displays the XRD patterns of the as prepared samples, which are the Kaol, C-Kaol, and PS- Kaol, respectively. For the C-Kaol, the diffraction peaks are almost disappeared, and are replaced by a broad background which is the characteristic of metakaolinite. After hydrothermally treated at 200 °C for 48 h, the (001), (020), and (110) reflections are reappeared clearly which illustrate that the metakaolinite is rehydrated and transformed to Kaol again. However, the reflections in the range of 30–40° (2θ) degree of PS-Kaol are broad compared with Kaol, indicating that PS-Kaol is poorly crystallized.

Figure 1b shows FTIR spectra of original kaolinite, C-Kaol, and PS-Kaol samples. In comparison with spectrum of original kaolinite, the hydroxyl peaks in the range 3700–3600 cm^−1^ are invisible for the C-Kaol, and the bands related to Si-O vibrations in the ranges 1110–1000 cm^−1^ [34] were visibly broadened. The bands of Al-O-Si vibration at 795, 750 cm^−1^ [34] are also broadened and the peak of octahedral aluminum at 912 cm^−1^ [35] is disappeared. These results indicate that the kaolinite has totally changed to amorphous metakaolinite after 2 h calcined at 600 °C. While, after hydrothermal treatment, a broad band of hydroxyl groups in the 3700–3600 cm^−1^ was appeared for the PS-Kaol. Furthermore, the broadened Si-O vibrations band becomes sharper and the octahedral aluminum at 912 cm^−1^ is appeared again compared with C-Kaol. The above changes for the Kaol, C-Kaol, and PS-Kaol reveal that after hydrothermal treatment the calcined metakalinite is rehydrated and somewhat changed back to kaolinite with a low crystallization.

To further characterize the surface property of the prepared samples, the binding energy of Al_*2p*_ and Si_*2p*_ for Kaol, C-Kaol, and PS-Kaol were determined by XPS (Fig. 1c, d). The observed chemical structure of Si and Al in the samples changed after calcination and hydrothermal treatment. The binding energy of Si_*2p*_ and Al_*2p*_ of C-Kaol are increased by 0.16 and 0.67 ev compared with that of Kaol, respectively. After hydrothermal treatment, the binding energy of Al_*2p*_ almost keep the same with that of C-Kaol, while the Si_*2p*_ further increased by 0.26 ev. These results show that chemical environment of Al and Si are changed under the calcined and hydrothermal treatment. The Si_*2p*_/Al_*2p*_ area ratios and the corresponding Si/Al atomic ratios obtained for all samples are list in Fig. 1c, d. Note that both ratios for C-Kaol are quite similar to that of Kaol. This illustrate that calcined treatment does not change the distribution of Si and Al on the samples surface. While a remarkable decrease is found in the Si/Al atomic ratios and Si_2*p*_/Al_2*p*_ area ratios of PS-Kaol (1.05 and 1.68) with respect to that of Kaol (1.12 and 1.78). This suggests that the hydrothermal treatment promotes an aluminum enrichment of kaolinite surface. Some research observed the same phenomenon when the coal gangue was mechanically grinding modified, and proposed that this aluminum enrichment new surface exhibited improved chemical reactivity [36].

**Fig. 2:**
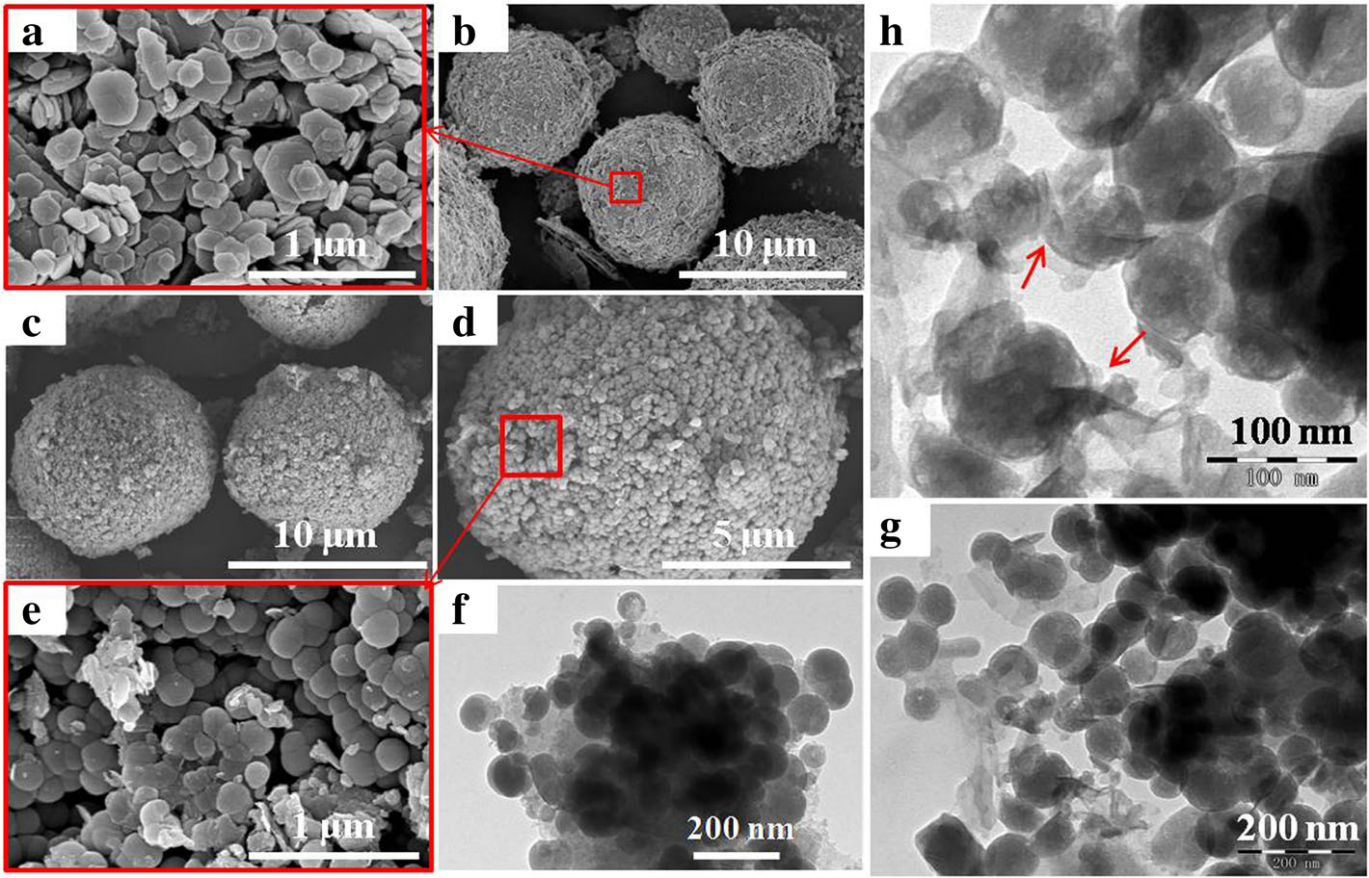
SEM images of samples at different magnifications. **a**, **b** Kaol. **c**–**e** Hydrothermal treatment metakaolinite (PS-Kaol). **f**–**h** TEM images of PS-Kaol

The morphologies of Kaol and PS-Kaol measured by SEM and TEM are presented in Fig. 2. The spray-dried Kaol aggregation shows microspheres structure with diameter of ~ 10 μm (Fig. 2a), which consists of numerous pseudo-hexagonal layer particles (Fig. 2b). There are lots of interparticle space in the Kaol microspheres which allow the water molecules easily to pass through the whole microsphere. For calcined samples, the morphology are almost the same with spray-dried Kaol (not list here). During the calcined treatment, Al in the octahedral sheet changes from a six- to fourfold coordination, while Si remains in fourfold coordination in the tetrahedral sheet, and the Kaol retains its layered structure [33]. After hydrothermal treatment, the C-Kaol changed to pomegranate-like structure microspheres. Figure 2c, d shows the whole image of the PS-Kaol with diameter of ~ 10 μm which is almost the same with the diameter of Kaol aggregation. The SEM image (Fig. 2e) with higher magnification shows detailed information that the PS-Kaol is constituted of many nanospheres. These nanospheres with a well-defined outline coalesce together and formed pores within the pomegranate-like superparticle. These results illustrate that the pseudo-hexagonal layer Kaol particles transformed to nanospheres without collapse of the spray-dried aggregation microspheres under the hydrothermal treatment. The XRD results revealed that these nanospheres were Kaol (Fig. 1), and other studies also recognized this kind of spheres as Kaol [22]. From the TEM micrographs (Fig. 2f–h), it was observed that these nanospheres with an average diameter of 20 nm were constructed with ultrathin flakes. Figure 2h revealed the ultrathin flakes scrolling around the nanospheres. These results imply that kaolinite nanospheres are formed by the aggregated thin kaolinite flakes and growing with the continues covering of thin flakes. Some researchers pointed out that the formation of kaolinite followed a dissolution-precipitation process [22, 37]. In the present paper, the formation of PS-Kaol may obey the following process. Firsly, the pseudo-hexagonal Kaol particles were agregated to form ball aggregation during the spray drying and were activated by the followed calcined treatment. The C-Kaol plate particles were dissolved under hydrothermal treatment and precipitated in situ to form ultrathin flakes. Subsequently, the growing flakes were transformed to spherical particles due to the tension of water.

**Fig. 3:**
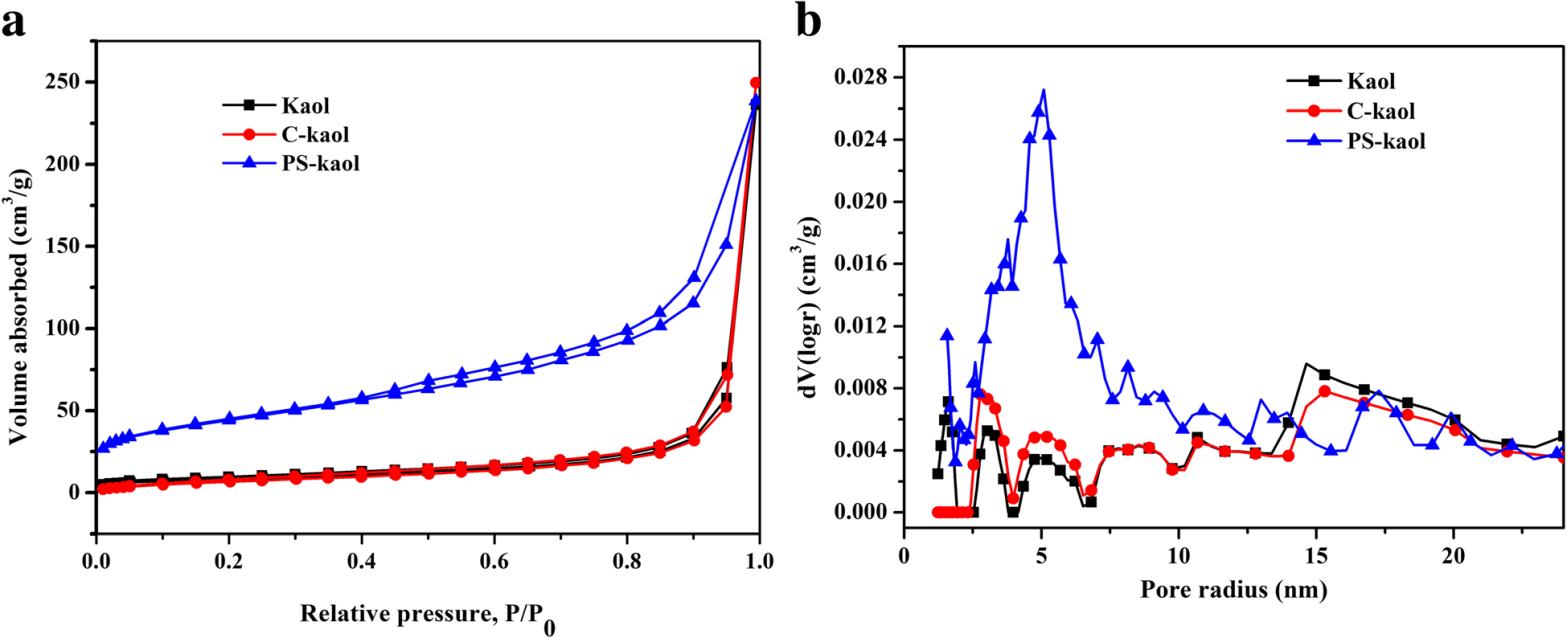
Nitrogen adsorption-desorption isotherms (**a**) and DFT pore size distribution curves (**b**) of Kaol, calcined Kaolinite (C-Kaol), and the hydrothermal treatment calcined Kaolinite (PS-Kaol)

The surface area and pore structure of Kaol, C-Kaol, and PS-Kaol are investigated by nitrogen adsorption-desorption, and the results are shown in Fig. 3. It can be seen that the isotherm of Kaol is very similar to a type II isotherm indicating that Kaol is a macroporous aggregate. After calcined, the isotherm of C-Kaol is almost the same with that of Kaol. However, the hydrothermal treatment shows a strong effect on the structure of the resulting samples. The adsorption amount of N_2_ for the PS-Kaol is sharply increased. The nitrogen adsorption-desorption isotherm of PS-Kaol shows a characteristic of type IV with an apparent hysteresis loop at relative pressure ranging from 0.40 to 0.99, suggesting the presence of abundant mesopores. The pore size distribution curves (Fig. 3b) of the samples evaluated using density functional theory (DFT) model show a pore size distribution at region of 2.0–10.0 nm with a maximum peak at 5.0 nm. The BET-specific surface area for the PS-Kaol is 157.1 m^2^ g^−1^, which is much higher than that of Kaol (29.3 m^2^ g^−1^) and C-Kaol (27.5 m^2^ g^−1^).

## MB Adsorption Performance

### Influence of Contact Time

**Fig. 4:**
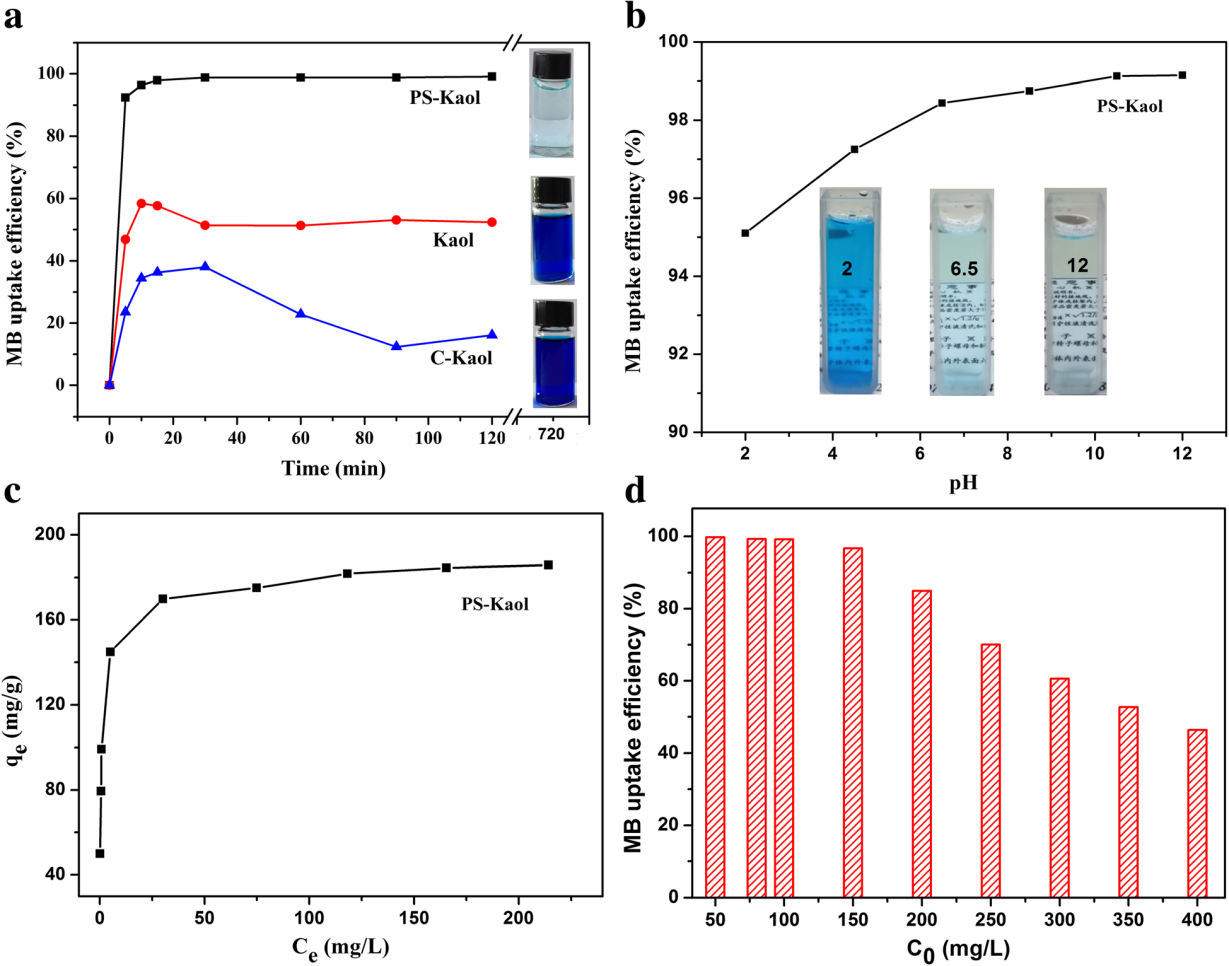
**a** Sorption rates of Kaol, C-Kaol, and PS-Kaol samples, 25 °C. **b** MB uptake efficiency of PS-Kaol sample at various initial solution pH. **c** Adsorption isotherms of PS-Kaol sample. **d** MB uptake efficiency of PS-Kaol sample at different initial MB concentration

The adsorptive capacity of the samples was evaluated using MB as typical indicator. Figure 4a shows the MB evolution with the contact time. The removal rate of MB from aqueous solutions by PS-Kaol was quickly reached to over 92% for only 5 min, and then increased slightly with contact time and reached to 99.1% for 120 min. For Kaol, the highest removal rate (57.6%) was achieved at 10 min, and then slightly reduced to 52.3% with the elongation contact time. For C-Kaol, the highest removal rate (38.1%) was achieved at 30 min and then sharply reduced to 16.1% with the increased contact time. This comparison results showed that the hydrothermal treatment greatly improved the adsorption ability of PS-Kaol and increased the adhesion affection between PS-Kaol particles surface and MB molecules.

### Influence of pH

As shown in Fig. 4b, the uptake efficiency of the PS-Kaol for MB increased from 95.10 to 99.15% with increasing the pH value from 2 to 12. Similar observations have been reported on MB adsorption on modified mesoporous clay [38] and kaolin [39]. The pH effect on dye adsorption can be explained by electrostatic interaction between adsorbent and dye molecules. The MB is a well-known cationic dye and with positive charge in solution, while the surface charge of kaolinite is strongly influenced by the pH of the solution. For kaolinite, as the pH of the solution increases, the number of negatively charged sites increases and the number of positively charged sites decreases [40]. Therefore, the extent of dyes adsorbed on kaolinite tends to increase with the increase of pH values. For PS-Kaol, the uptake efficiency for MB is also increased with the increase of pH values, while during the wide pH range (from 2 to 12) the uptake efficiency for MB is just slightly increased from 95.10 to 99.15%. Similar results were obtained for the remove of MB by acid treated kaolinite [15]. This adsorption behavior of the PS-Kaol at various pH suggest that it can be potentially applied in a wide pH range.

### Influence of Initial MB Concentration

The effect of an initial dye concentration on adsorption of MB dye was determined by preparing different concentrations of dye from 50 to 400 mg/L. The obtained sorption isotherms (Fig. 4c) reveal that the MB adsorption capacity sharply increases from 49.8 to 184.9 mg/g indicating significant potential of PS-Kaol for cationic dyes adsorption. Furthermore, Fig. 4d shows that the uptake efficiency of PS-Kaol exceeds 96% at initial MB concentrations ranging from 50 to 150 mg/L and then slowly dropped to the value (46%) at 400 mg/L, suggesting the high applicability of PS-Kaol in a broad concentration range of MB in wastewater.

## Adsorption Kinetic and Isotherm Models

To further investigate the adsorption characteristics of PS-Kaol toward MB dye, the adsorption kinetic (pseudo-first-order and pseudo-second-order) and isotherm (Langmuir and Freundlich equations) models are proposed according to the experimental data (Fig. 4). The corresponding equations are given:

4 $$ \mathrm{Pseudo}\hbox{-} \mathrm{first}\hbox{-} \mathrm{order}:\kern0.5em \ln \left({q}_{\mathrm{e}}\kern0.5em -\kern0.5em {q}_{\mathrm{t}}\right)\kern0.5em =\kern0.5em \ln {q}_{\mathrm{e}}\kern0.5em -\kern0.5em {K}_1t $$

5 $$ \mathrm{Pseudo}\hbox{-} \mathrm{second}\hbox{-} \mathrm{order}:\kern0.5em \frac{t}{q_{\mathrm{t}}}\kern0.5em =\kern0.75em \frac{1}{K_2{q_{\mathrm{e}}}^2}\kern0.5em +\kern0.5em \frac{t}{q_{\mathrm{e}}} $$

6 $$ \mathrm{Langmuir}:\kern0.5em \frac{C_e}{\ {q}_e}=\frac{1}{K_L{q}_m}\kern0.5em +\frac{C_e}{q_m} $$

7 $$ \mathrm{Freundlich}:\kern0.5em {lnq}_e={lnK}_F+\frac{1}{n}{lnC}_e $$

where *K*_1_ (1min^− 1^) and *K*_2_ (g/mg/min) are the pseudo-first-order and pseudo-second-order rate constants, respectively. *q*_m_ (mg/g) and *K*_L_ (L/mg) are Langmuir isotherm coefficients; *K*_F_ (mg/g) and *n* are Freundlich constants.

**Fig. 5:**
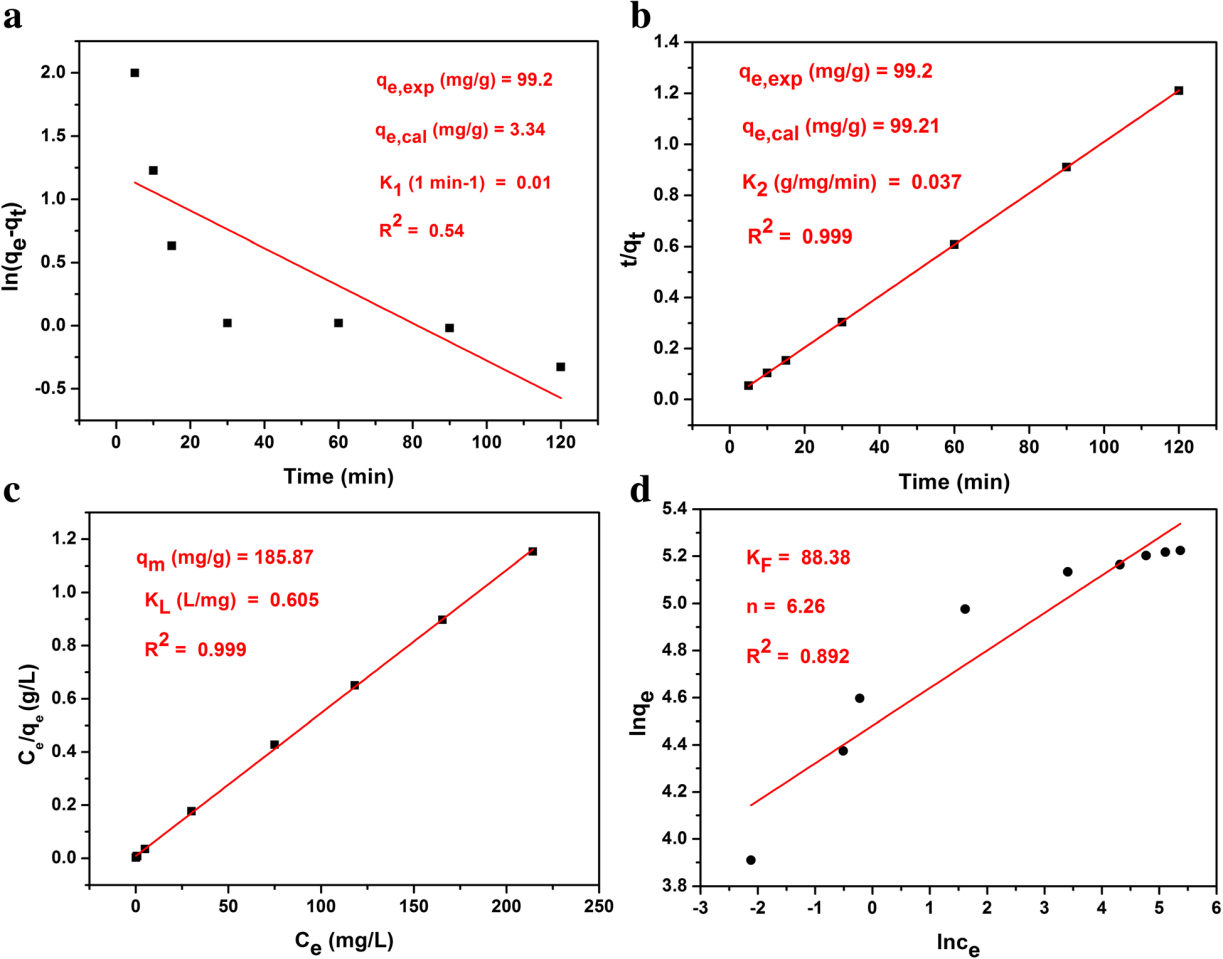
**a**, **b** Pseudo-first-order and pseudo-second-order kinetic models for the adsorption of MB dye onto PS-Kaol, respectively. **c**, **d** Langmuir and Freundlich isotherm models for the adsorption of MB dye onto PS-Kaol, respectively

Adsorption kinetics was carried out to evaluate the rate and mechanism of the dye molecules transfer from the liquid solution onto the PS-Kaol surface. The data and fittings of pseudo-first-order and pseudo-second-order kinetic models were shown in Fig. 5a, b, respectively. The values of *R*^2^ of pseudo-first-order and pseudo-second-order models were 0.54 and 0.999, respectively. Furthermore, the values of *q*_e_,cal (99.21) for pseudo-second-order models also appeared to be very close to the experimentally observed values of *q*_e_,exp. (99.2). These results obviously show that the adsorption of MB onto PS-kaol is dominated by the pseudo-second-order adsorption mechanism, and indicate the chemisorption nature of MB on PS-Kaol surface. The adsorption rate constant *K*_2_ of PS-Kaol toward MB is 0.037 g/(mg min) according to the pseudo-second-order kinetic model.

**Fig. 6:**
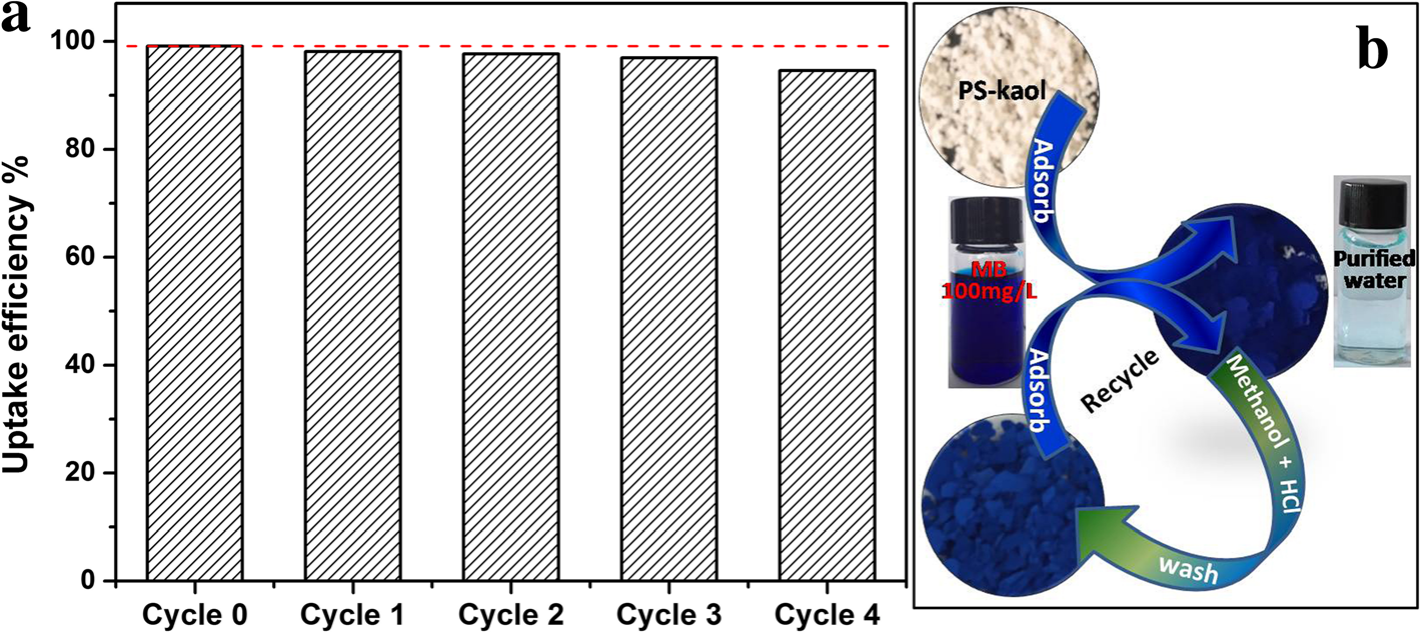
**a** Different reusability cycles of PS-Kaol for removed MB. **b** Schematic image of the application and regeneration of PS-Kaol for water purification

The Langmuir and Freundlich isotherm models were used for the adsorption analysis and the linear fitting results were displayed in Fig. 6c, d, respectively. The data fits better to the Langmuir isotherm with a correlation coefficient *R*^2^ value of 0.999 (Fig. 5c) than to the Freundlich isotherm with a correlation coefficient of 0.892 (Fig. 5d), indicating the monolayer adsorption of MB on the PS-Kaol surface. The *q*_m_ value of MB on PS-Kaol was 185.87 mg/g, close to the experimental data (184.9 mg/g). Based on the characterization, adsorption performance, and adsorption isotherm and kinetics model analysis, the enhanced adsorption property of PS-Kaol could be attributed to the improved highly specific surface area propriety. Additionally, the hierarchical mesoporous structure of nanoparticles was also helpful to the diffusion and transport of MB molecules (Fig. 3).

Reusability potential of sorbent is a significant factor for its practical uses. The attached pollutants could be dislocated by inserting proper solvent molecules [41, 42]. In this study, the utilized PS-Kaol was regenerated by the washing with methanol solution containing 0.1 mM HCl for the next adsorption. As can be observed in Fig. 6a, after 4 cycles, the uptake efficiency of adsorbent reduced slightly compared with the remove efficiency 99.1% of original PS-Kaol, and the dye elimination after 1 and 4 cycles was 98.09% and 94.61%, respectively. So, with the increase of the regeneration cycles, the recovered adsorption capacity was gradually diminishing. As illustrated in Fig. 6b, after adsorb MB dye, the white color of PS-kao changed to dark blue, and the contaminated water was remedied to clean water. The used dark blue PS-kao was regenerated by the dislodgement of MB dye through washing with methanol + HCl solution and changed as light blue color. This implied that the adsorbed MB days were not thoroughly dislodged by solvent washing, and was the reason for gradually diminished adsorption capacity of recycled PS-Kao. Some researchers also observed the successive adsorption capacity decrease when recycle adsorbent by solvent washing method [4, 42]. Here, the interesting result is that the solvent desorption regeneration of the PS-Kaol adsorbent could retain the high removal efficiency (recovered more than 95% adsorption capacity) during four successive cycles. Therefore, PS-Kaol with an excellent adsorption performance and regeneration property can be effectively employed for the dye removal from wastewater.

## Conclusions

In summary, pomegranate-like Kaol hierarchical structures were successfully prepared through calcined-hydrothermal approach using purified kaolin as starting material. The results obtained indicate that the C-Kaol plate particles are dissolved under hydrothermal condition and precipitated to ultrathin flaks which aggregated to form Kaol nanospheres due to the tension of water. PS-Kaol with high specific surface area and abundant mesopores shows excellent adsorption performance with high uptake efficiency to MB under broad pH conditions, fast sorption kinetics, and efficient sorbent regeneration. Thus, the PS-Kaol shows good application prospects for wastewater treatments and environmental remediation. This also provides an environment friendly tailoring technique to prepare clay-based functional nanostructure materials.

## Abbreviations

C-Kaol: Calcined kaolinite
Kaol: Kaolinite
MB: Methylene blue
PS-Kaol: Pomegranate-like Kaolinite spheres
